# A Concomitant Muscle Injury Does Not Worsen Traumatic Brain Injury Outcomes in Mice

**DOI:** 10.3389/fneur.2018.01089

**Published:** 2018-12-11

**Authors:** Mujun Sun, Rhys D. Brady, Chris van der Poel, Danielle Apted, Bridgette D. Semple, Jarrod E. Church, Terence J. O'Brien, Stuart J. McDonald, Sandy R. Shultz

**Affiliations:** ^1^Department of Medicine, The Royal Melbourne Hospital, The University of Melbourne, Melbourne, VIC, Australia; ^2^Departments of Neuroscience and Medicine, Central Clinical School, Monash University, Melbourne, VIC, Australia; ^3^Department of Physiology, Anatomy and Microbiology, La Trobe University, Melbourne, VIC, Australia

**Keywords:** polytrauma, weight-drop, cardiotoxin, neuroinflammation, cytokines

## Abstract

Traumatic brain injury (TBI) often involves multitrauma in which concurrent extracranial injury occurs. We previously demonstrated that a long bone fracture exacerbates neuroinflammation and functional outcomes in mice given a TBI. Whether other forms of concomitant peripheral trauma that are common in the TBI setting, such as skeletal muscle injury, have similar effects is unknown. As such, here we developed a novel mouse multitrauma model by combining a closed-skull TBI with a cardiotoxin (CTX)-induced muscle injury to investigate whether muscle injury affects TBI outcomes. Adult male mice were assigned to four groups: sham-TBI + sham-muscle injury (SHAM); sham-TBI + CTX-muscle injury (CTX); TBI + sham-muscle injury (TBI); TBI + CTX-muscle injury (MULTI). Some mice were euthanized at 24 h post-injury to assess neuroinflammation and cerebral edema. The remaining mice underwent behavioral testing after a 30-day recovery period, and were euthanized at 35 days post-injury for post-mortem analysis. At 24 h post-injury, both TBI and MULTI mice had elevated edema, increased expression of GFAP (i.e., a marker for reactive astrocytes), and increased mRNA levels of inflammatory chemokines. There was also an effect of injury on cytokine levels at 35 days post-injury. However, the TBI and MULTI mice did not significantly differ on any of the measures assessed. These initial findings suggest that a concomitant muscle injury does not significantly affect preclinical TBI outcomes. Future studies should investigate the combination of different injury models, additional outcomes, and other post-injury time points.

## Introduction

Traumatic brain injury (TBI) is caused by external forces applied to the brain and is a common consequence of motor vehicle accidents, sports, slips, and falls, industrial accidents, and war (1). TBI is a leading cause of death and disability worldwide and involves a complex pathophysiology that includes primary and secondary injury pathways (1, 2). Primary injury involves direct tissue damage caused by mechanical forces at the moment of impact. The primary injury also initiates a series of secondary injury processes, which include neuroinflammation (3–5), oxidative stress (6, 7), apoptosis (8), and further blood-brain barrier (BBB) damage (9). These secondary injuries occur within minutes to hours after TBI, and may persist into chronic stages and contribute to neurodegeneration (1, 2).

Due to the high-impact nature of TBI-inducing accidents, approximately one-third of TBI patients sustain a concomitant extracranial trauma, otherwise known as multitrauma or polytrauma (10–14). For example, significant skeletal muscle injury often occurs in the presence of TBI (15, 16). Muscle injury involves degeneration and regeneration of myofibers, during which the inflammatory process is of particular importance (17, 18). Human studies have found that neutrophils are rapidly upregulated in circulation and at the lesion site after muscle injury (19). Using cardiotoxin (CTX) to induce a muscle injury in an experimental mouse model, neutrophils were observed to accumulate in the injured muscle for at least 3 days after injury, and macrophages began to accumulate at ~3 days post-injury (20). Pro-inflammatory cytokines have also been found to be up-regulated in injured muscle during the first week after CTX injection in mice (21).

Notably, many of the immune cells and cytokines involved in skeletal muscle injury are also important modulators in TBI pathology, and BBB disruption in TBI may facilitate the ability of peripheral factors to enter the brain (22, 23). However, most pre-clinical TBI studies have utilized independent “single-hit” injury models that do not account for concomitant injuries, and their potential interactive pathophysiologies, that frequently occur with TBI (22–24). Of relevance, we have previously demonstrated that a concurrent long bone fracture worsens TBI outcomes in mice, and that this was associated with an exacerbated and prolonged neuroinflammatory response (3, 5). It therefore stands to reason that other concomitant extracranial injuries, such as muscle injury, may also affect TBI pathobiology and outcomes in a similar manner. Therefore, here we developed a mouse model of multitrauma that combined a weight-drop induced TBI and a CTX-induced muscle injury, to assess whether a concurrent muscle injury affected TBI outcomes.

## Materials and Methods

### Mice

A total of 91 C57BL/6 male mice were obtained from the Australia Animal Resource Centre, and housed individually under a 12 h light/dark cycle with access to food and water *ad libitum*. Mice were 12 weeks of age at the time of injury. All procedures were approved by The Florey Institute of Neuroscience and Mental Health Animal Ethics Committee, and were within the guidelines of the Australian Code of Practice for the Care and Use of Animals for Scientific Purposes by the Australian National Health and Medical Research Council.

### Experimental Groups

Mice were randomly assigned to one of four experimental groups: sham-TBI + sham-muscle injury (SHAM, *n* = 18); sham-TBI + CTX-muscle injury (CTX, *n* = 20); TBI + sham-muscle injury (TBI, *n* = 27); TBI + CTX-muscle injury (MULTI, *n* = 26). Mice that died immediately after TBI (*n* = 14, 26% mortality rate) were excluded from the study. A portion of mice (SHAM, *n* = 6; CTX, *n* = 7; TBI, *n* = 6; MUTLI, *n* = 6) were euthanized at 24 h post-injury and underwent post-mortem analysis. The remaining mice (SHAM, *n* = 12; CTX, *n* = 13; TBI, *n* = 14; MULTI, *n* = 13) underwent behavioral testing after a 30-day recovery period and were euthanized at 35 days post-injury for post-mortem analysis. These recovery times are consistent with our previous study that investigated the effects of a concomitant fracture on TBI in mice (3).

### The Weight-Drop Model of TBI

A weight-drop device was used to induce a closed-skull TBI as previously described (3, 25). The weight-drop device consisted of a guided- and weighted-rod with a blunt silicone-covered impact tip (3 mm diameter). Each mouse was placed in an anesthesia induction chamber containing 4% isoflurane for 2 min. Once anesthetized, mice were placed in a nose cone that maintained the anesthetic (2% isoflurane) for 3 min and administered 0.05 mg/kg of buprenorphine analgesic subcutaneously. Mice were given a sham or CTX muscle injury as described below, then a 2 cm incision was made along the midline of the scalp to reveal the intact skull. The mouse then removed from the nose cone, stabilized on the weight-drop device platform, the 215 g weighted-rod was released from a distance of 2.5 cm, and the impact tip made contact between the sagittal and coronal suture of the right hemisphere. The rod was retracted immediately after the impact occurred, and the scalp incision was sutured. The sham TBI procedure for the SHAM and CTX groups was identical to that described for the TBI procedure, except the weighted-rod was not released so that no impact occurred. Duration of apnea, loss of consciousness (i.e., hind-limb withdrawal to toe pinch), and self-righting reflex (latency to self-right) were recorded as indicators of acute injury severity (Table 1) (3, 26).

**Table 1:** Acute injury measures.

	**SHAM**	**CTX**	**TBI**	**MULTI**
Apnea (s)	0.0	0.0	12.7 ± 1.5^*^	15.7 ± 1.4^*^
Hindlimb reflex (s)	45.1 ± 4.0	45.9 ± 4.6	142.5 ± 11.0^*^	124.1 ± 13.4^*^
Self-righting reflex (s)	56.1 ± 5.2	57.5 ± 5.3	188.1 ± 12.8^*^	166.3 ± 15.1^*^

*TBI and MULTI groups had significantly longer apnea, unconsciousness (i.e., hindlimb withdrawal reflex to toe pinch), and self-righting reflex times (seconds ± SEM) than SHAM and CTX groups. There were no statistically significant differences between TBI and MULTI*.

* *greater than SHAM and CTX, p < 0.001*.

### The CTX Model of Muscle Injury

To induce a muscle injury, mice in CTX and MULTI groups received a single intramuscular injection of CTX (50 μl of 10 μM solution in sterile saline; Sigma-Aldrich, Castle Hill, NSW, Australia) which was delivered percutaneously into the right hamstring (27). Mice in the SHAM and TBI groups received a single intramuscular injection of vehicle (50 μl sterile saline). CTX induces depolarization and contraction of muscle cells, and consequently damages the structure of cell membranes (28, 29). It is one of most reliable muscle injury models, and results in physiological events that occur in human muscle injury, including inflammation (18, 21, 29).

**Figure 1:**
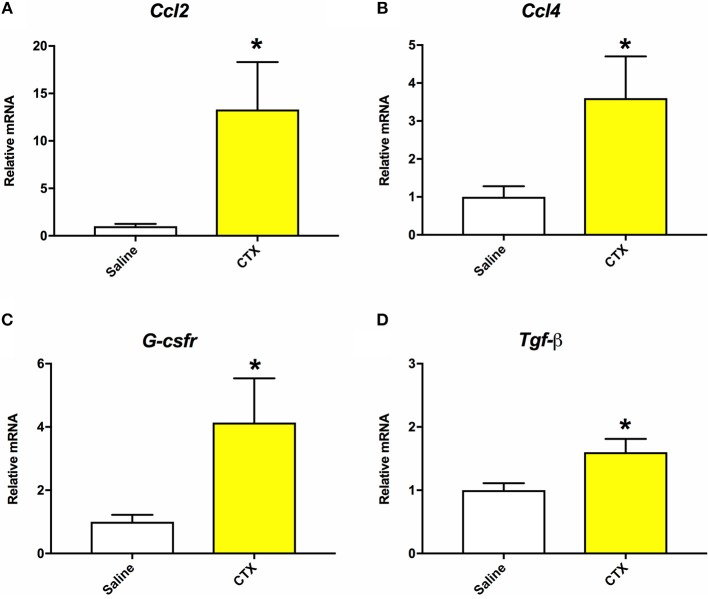
Mice that were injected with CTX into the right hamstring had significantly increased mRNA levels of *Ccl*2 **(A)**, *Ccl*4 **(B)**, *G-csfr* **(C)**, and *Tgf-*β **(D)** in the hamstring muscle tissue compared to saline injected mice at 24 h post-injury. There were no statistically significant differences on mRNA levels of *Il-1*β or *Tnf-*α. *different than saline, *p* < 0.05.

As detailed below in section Quantitative Real-Time PCR (RT-qPCR), muscle tissue was collected from mice that were injected with either saline or CTX and euthanized at 24 h post-injury. RT-qPCR was performed to assess gene expression of inflammatory markers as indicators of muscle injury in the CTX injected mice (30). As shown in Figure 1, mice injected with CTX had significantly elevated mRNA levels of *Ccl2, Ccl4, G-csfr*, and *Tgf-*β in the injected hamstring compared to saline injected mice (*p* < 0.05). Furthermore, although not done in a quantitative manner, daily health monitoring by individuals who were blinded to the experimental groups indicated that the mice injected with CTX consistently displayed right hindlimb abnormalities for 2–3 days after the injection, whereas those injected with saline did not.

### Cerebral Edema Analysis

As previously described (3, 5), brain water content was assessed as an indicator of cerebral edema. Mice were anesthetized with isoflurane and decapitated at 24 h post-injury. The cortex adjacent to the impact site was rapidly dissected, immediately weighed (wet weight), and then dried at 100°C for 24 h. The tissue was again weighed (dry weight), and the following formula was used to determine brain water content: water content (%) = (wet weight–dry weight)/wet weight (5).

### Automated Capillary Western Blots

Capillary Western blots were used to assess protein expression of markers indicative of different immune cells. Mice were anesthetized and decapitated at either 24 h or 35 days post-injury, and the ipsilateral cortex directly under the impact site was dissected and rapidly frozen. The frozen brain tissue was homogenized in radioimmunoprecipitation assay (RIPA) buffer with protease and phosphatase inhibitors. The protein concentration of the lysates was quantified using a BCA Protein Assay Kit (Thermo Scientific Pierce Biotechnology, San Jose, United States) and Benchmark Plus Microplate Spectrophotometer (Bio-Rad, Hercules, United States). Capillary Western analyses were performed using the WES^TM^ Simple Western System (ProteinSimple, United States) according to the instructions provided. Briefly, samples were diluted with sample buffer, combined with Fluorescent Master Mix, and heated at 95°C for 5 min for denaturation. The denatured samples, blocking reagent, primary antibodies, HRP-conjugated secondary antibodies and chemiluminescent substrate were then loaded into a designated assay plate, and processed for automated separation electrophoresis and immunodetection (31). A biotinylated ladder was also loaded for each assay. Individual assays were performed for glial fibrillary acidic protein (GFAP; 1:1,000, Dako #Z0334; samples loaded at 0.2 μg/μl), a marker for astrogliosis (32); cluster of differentiation (CD) 68[ED1] (1:10, Abcam #ab31630; samples loaded at 2 μg/μl), a marker for microglia/macrophages (33, 34); and myeloperoxidase (MPO; 1:10, Abcam #ab9535; samples loaded at 2 μg/μl), a marker for neutrophils, and monocytes to a lesser extent (35). Glyceraldehyde 3-phosphate dehydrogenase (GAPDH; 1:100, Santa Cruz Biotechnology #sc-47724) or β-actin (1:50, Abcam #ab8227) were used as loading controls.

### Quantitative Real-Time PCR (RT-qPCR)

RT-qPCR was used to assess gene expression of inflammatory markers in brain and muscle tissue. Mice were anesthetized with isoflurane and decapitated at either 24 h or 35 days post-injury. For the examination of brain tissue, the ipsilateral cortex directly under the impact site was rapidly dissected, frozen in liquid nitrogen, and stored at −80°C. For the examination of muscle tissue, 100 mg of tissue was rapidly dissected from the injection site in the right hamstring, frozen in liquid nitrogen, and stored at −80°C. Frozen brain and muscle tissue was homogenized with PureZOL^TM^ RNA isolation reagent, and total RNA were extracted using the Aurum^TM^ Total RNA Fatty and Fibrous Tissue Kit (Bio-Rad, Hercules, USA) (36). RNA for each sample (brain = 600 ng, muscle = 1,000 ng) was then reverse transcribed to cDNA.

The primer sequences for the inflammatory genes of interest are summarized in Table 2 (30, 37). qPCR was run using the iCycler iQ Multi-Color RT-PCR detection system (using SsoFast^TM^ EvaGreen, Bio-Rad, Hercules, USA). To establish specificity of DNA products, melt-curve analysis was performed. Cycle threshold (Ct) values were collected for analysis, and data was normalized to internal control (brain = *Gapdh;* muscle = β*2-microglobulin*). Relative quantification of genes of interest mRNA expression was determined using the 2^−ΔΔCt^ method (5).

**Table 2:** RT-qPCR markers.

**Gene**	**Forward primer sequence 5^**′**^-3^**′**^**	**Reverse primer sequence 5^**′**^-3^**′**^**
**BRAIN**		
*Ccl2*	CAAGATGATCCCAATGAGTAG	TTGGTGACAAAAACTACAGC
*Ccl4*	GGTATTCCTGACCAAAAGAG	TCCAAGTCACTCATGTACTC
*Ccl12*	CAGTCCTCAGGTATTGGCTGGA	TCCTTGGGGTCAGCACAGAT
*Il-1β*	TGTAATGAAAGACGGCACACC	TCTTCTTTGGGTATTGCTTGG
*Tnf-α*	CCCTCACACTCAGATCATCTTCT	GCTACGACGTGGGCTACAG
*Il-6*	GAGCCCACCAAGAACGATAG	GGTTGTCACCAGCATCAGTC
*Cd36*	GGACATTGAGATTCTTTTCCTCTG	GCAAAGGCATTGGCTGGAAGAAC
*Il-4rα*	CAGATCCCAGATACTCACTG	AGTAACAGAACAGGCAAAAC
*Tgf-β1*	TGATACGCCTGAGTGGCTGTCT	CACAAGAGCAGTGAGCGCTGAA
*Gapdh*	ATGACATCAAGAAGGTGGTG	CATACCAGGAAATGAGCTTG
**MUSCLE**		
*G-csfr*	ACAAAGCAGGGACCTCTTCA	ATGGTGTTAAGGTCTTGGGC
*Tgf-β*	ATTCCTGGCGTTACCTTGG	CCTGTATTCCGTCTCCTTGG
*Ccl2*	CAAGATGATCCCAATGAGTAG	TTGGTGACAAAAACTACAGC
*Ccl4*	GGTATTCCTGACCAAAAGAG	TCCAAGTCACTCATGTACTC
*Tnf-α*	CCCTCACACTCAGATCATCTTCT	GCTACGACGTGGGCTACAG
*Il-1β*	TCCATGAGCTTTGTACAAGG	GGTGCTGATGTACCAGTTGG
*β2-mg*	TTCACCCCCACTGAGACTGAT	GTCTTGGGGTCGGCCATA

*Inflammatory genes of interest for brain and muscle tissue analysis included chemokine (C-C motif) ligand (Ccl)2, Ccl4, Ccl12, interleukin-1β (Il-1β), Il-6, tumor necrosis factor-α (Tnf-α), Cd36, IL-4 receptor α (Il-4rα), granulocyte colony-stimulating factor receptor (G-csfr), and transforming growth factor-β1 (Tgf-β1). Glyceraldehyde 3-phosphate dehydrogenase (Gapdh) and β2-microglobulin (β2-mg) served as housekeeping genes*.

### Behavioral Testing

After a 30-day recovery period, a subset of mice underwent behavioral testing. With the exception of the rotarod, all tests were recorded by an overhead camera and objectively analyzed by Ethovision tracking software (EthoVision®, Noldus, United States). Testing was conducted by an investigator blinded to experimental conditions.

Open field was used to assess locomotor and anxiety-like behavior (26, 38). The open field was a circular arena (100 cm diameter) shielded by 30 cm high walls. A circular area (66 cm diameter) in the arena was defined as the middle field. The mouse was released in the center and allowed to explore for 5 min. Distance traveled, time in the middle field, and middle field entries were calculated.

Anxiety-like behavior was assessed using the elevated-plus maze (San Diego Instruments, USA) (38, 39). The maze consisted of two opposing opened arms and two opposing closed arms (30 cm × 6 cm) shaped like a “plus.” The closed arms were shielded by walls (15 cm high), while the opened arms were not. Each mouse was placed in the center of the maze facing an open arm and allowed to explore for 5 min. The proportion of time spent in the open arms was calculated to analyze anxiety-related behavior. The number of closed arms entries and total travel distance were calculated for the measurements of activity and motor function.

Motor function was assessed by the rotarod (Harvard Apparatus, United States) (26). The apparatus consisted of a rotating barrel (3 cm diameter) divided by walls into four equal lanes (5 cm width). Three trials were performed each day for two consecutive days. For each trial, the mouse was placed on the rotating barrel with a start speed of 0.0027 g, and the speed accelerated to 0.27 g over a 5 min period. The duration of time on the rotarod and the speed that the mouse was able to achieve were recorded.

Spatial cognition was assessed in Y-maze as previously described (26, 40). The Y-maze consisted of 3 arms (each 38 × 8 cm) shielded with walls (13 cm high) and adjoined in a Y-shape (San Diego Instruments, United States). At the distal end of each arm was an exterior visual cue. Each mouse underwent 15 min training in which one arm was blocked (novel arm), and the mouse was allowed to freely explore the other two arms after released from a start arm. After training, the mouse was given a 30 min interval time and then underwent a 5 min test in which the novel arm was unblocked, and the mouse was released in the same start arm. The time spent and the number of entries into the novel arm were quantified as measures of spatial cognition.

### Statistical Analysis

All outcomes were analyzed with SPSS 24.0 software (IBM Corp, USA) using a one-way analysis of variance (ANOVA). Bonferroni *post-hoc* comparisons were carried out when appropriate. Statistical significance was set as *p* ≤ 0.05.

## Results

**Figure 2:**
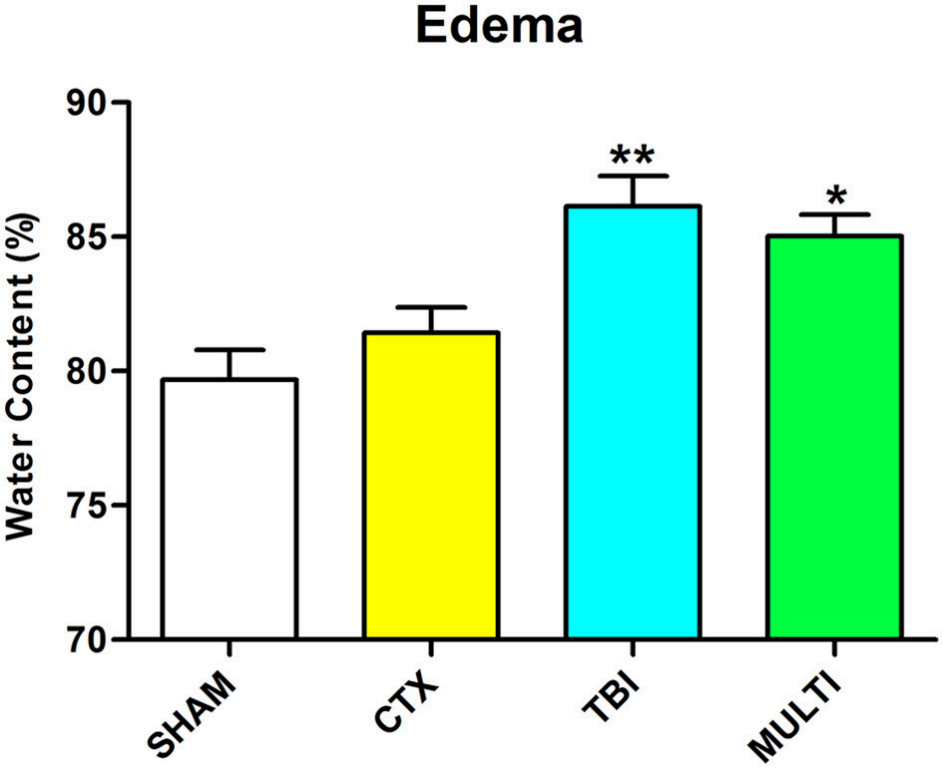
Brain water content was assessed as an indicator of cerebral edema. TBI and MULTI mice had significantly increased brain water content compared to the SHAM mice, and the TBI mice also had significantly increased brain water content compared to the CTX mice. *different than SHAM; **different than SHAM and CTX; *p* < 0.05. See Results for additional details.

Brain water content was assessed as an indicator of cerebral edema. ANOVA identified a significant injury effect on the measure of brain water content in the ipsilateral cortex at 24 h post-injury [*F*_(3, 16)_ = 9.003, *p* < 0.01; Figure 2]. *Post-hoc* analysis indicated that TBI and MULTI mice had significantly increased brain water content compared to the SHAM mice (*p* < 0.05), and the TBI mice also had significantly increased brain water content compared to the CTX mice (*p* < 0.05). There was no statistically significant difference between the TBI and MULTI groups on brain water content.

**Figure 3:**
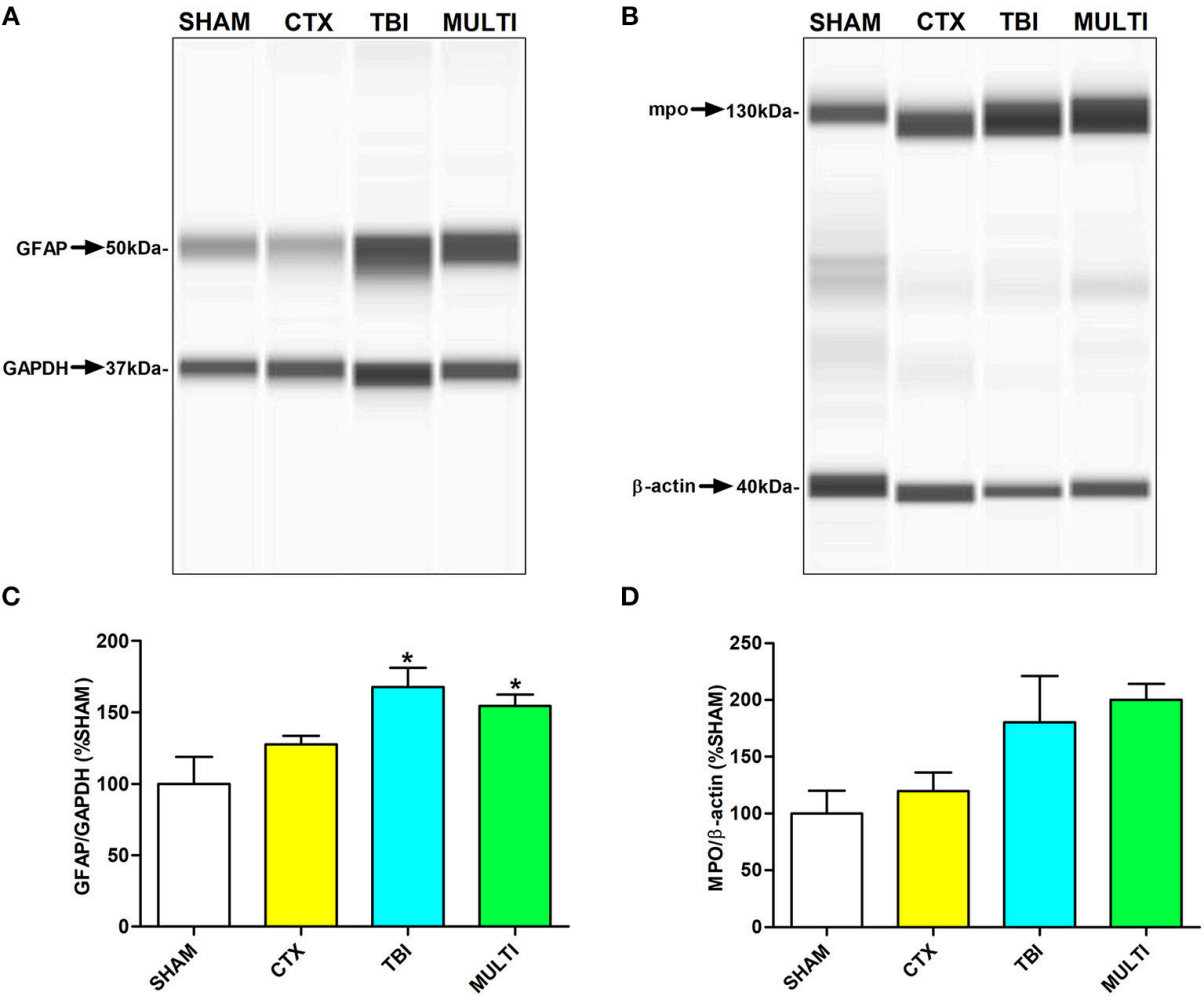
Automated capillary Western blots were used to quantify protein expression of markers related to different immune cells in the ipsilateral cortex. **(A)** Pseudo-gel image of representative blots of GFAP and GAPDH for each of the four groups. **(B)** Pseudo-gel image of representative blots of MPO and β-actin for each of the four groups. **(C)** At 24 h post-injury, TBI and MULTI mice had significantly increased levels of GFAP, a marker for reactive astrocytes, compared to SHAM. **(D)** At 24 h post-injury, there was a significant effect of injury on the measure of MPO (i.e., a marker for neutrophils. Although TBI and MULTI groups appeared to have similarly elevated levels of MPO, *post–hoc* analyses did not reach statistical significance after Bonferroni corrections. *different than SHAM, *p* < 0.05. See Results for additional details.

Automated capillary Western blots were used to quantify protein expression of markers related to different immune-related cells in the ipsilateral cortex. At 24 h post-injury, ANOVA identified a significant injury effect on expression of GFAP [i.e., a marker for astrogliosis; *F*_(3, 16)_ = 5.753, *p* < 0.01; Figures 3A,C] and MPO [i.e., a marker for neutrophils; *F*_(3, 16)_ = 3.613, *p* < 0.05; Figures 3B,D]. *Post-hoc* analyses indicated that TBI and MULTI mice had significantly increased levels of GFAP compared to SHAM (*p* < 0.05). Although TBI and MULTI groups appeared to have similarly elevated levels of MPO relative to the SHAM and CTX groups, *post-hoc* analyses did not reach statistical significance after Bonferroni corrections. There were no statistically significant findings on levels of CD68 at 24 h post-injury (data not shown). There were no statistically significant findings on any of the markers at 35 days post-injury (data not shown). The TBI and MULTI groups were not significantly different on any of the protein quantification measures.

**Figure 4:**
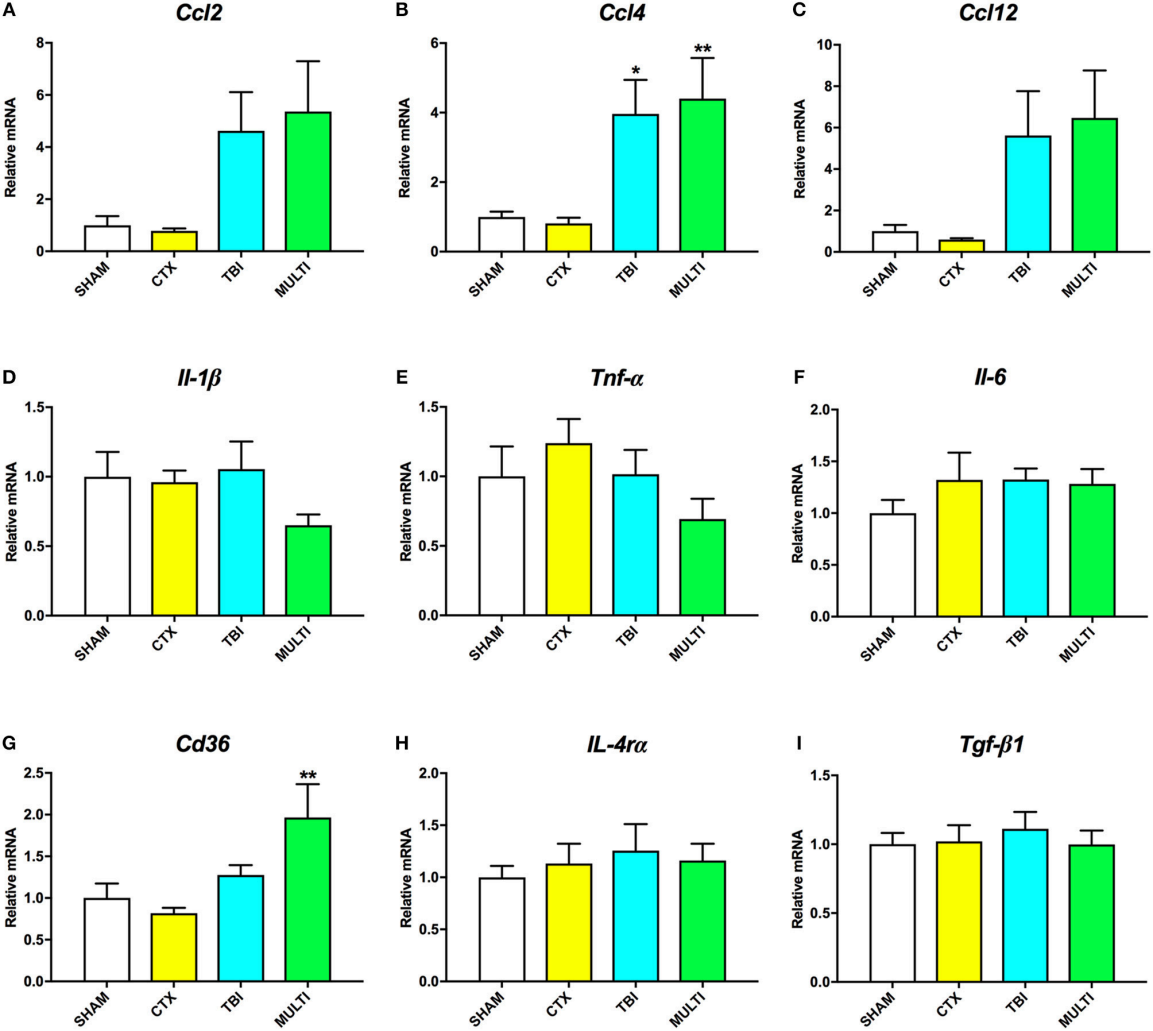
mRNA levels of pro- **(A–F)** and anti-inflammatory **(G–I)** cytokines in the ipsilateral cortex at 24h post-injury. There was a significant effect of injury on *Ccl*2 **(A)** and *Ccl*12 **(C)**. Although TBI and MULTI groups appeared to have similarly elevated levels of *Ccl*2 and *Ccl*12, *post-hoc* analyses did not reach statistical significance after Bonferroni corrections. **(B)** Both the TBI and MULTI groups had significantly increased *Ccl*4 mRNA levels compared to the CTX group, and that the MULTI group was also significantly higher than the SHAM group. **(G)** The MULTI group had significantly higher mRNA levels of *Cd*36 than both the CTX and SHAM groups. There were no significant differences on mRNA levels of *Il-1*β **(D)**, *Tnf-*α **(E)**, *Il-6* **(F)**, *Il-4r*α **(H)**, or *Tgf-*β*1* **(I)**. *different than CTX; **different than SHAM and CTX; *p* < 0.05. See Results for additional details.

RT-qPCR was used to assess mRNA levels of different pro- and anti-inflammatory cytokines in the ipsilateral cortex at 24 h and 35 days post-injury. At 24 h post-injury, ANOVA identified a significant effect of injury on the mRNA levels of the pro-inflammatory chemokines *Ccl2* [*F*_(3, 21)_ = 4.091, *p* < 0.05; Figure 4A], *Ccl*4 [*F*_(3, 21)_ = 6.606, *p* < 0.005; Figure 4B], and *Ccl*12 [*F*_(3, 21)_ = 4.126, *p* < 0.05; Figure 4C], as well as anti-inflammatory cell-surface marker *Cd*36 [*F*_(3, 21)_ = 5.257, *p* < 0.01; Figure 4G]. *Post-hoc* analyses found that both the TBI and MULTI groups had significantly increased *Ccl*4 mRNA levels compared to the CTX group (*p* < 0.05), and that the MULTI group was also significantly higher than the SHAM group (*p* < 0.05). Both the TBI and MULTI groups appeared to have elevated levels of *Ccl*2 and *Ccl*12, however *post-hoc* analyses did not reach statistical significance after Bonferroni corrections. For *Cd*36, the MULTI group had significantly higher mRNA levels than both the CTX and SHAM groups (*p* < 0.05). There were no significant differences on mRNA levels of *Il-1*β (Figure 4D), *Tnf-*α (Figure 4E), *Il-6* (Figure 4F), *Il-4r*α (Figure 4H), or *Tgf-*β*1* (Figure 4I). The TBI and MULTI groups were not significantly different on any of the 24 h RT-qPCR measures.

**Figure 5:**
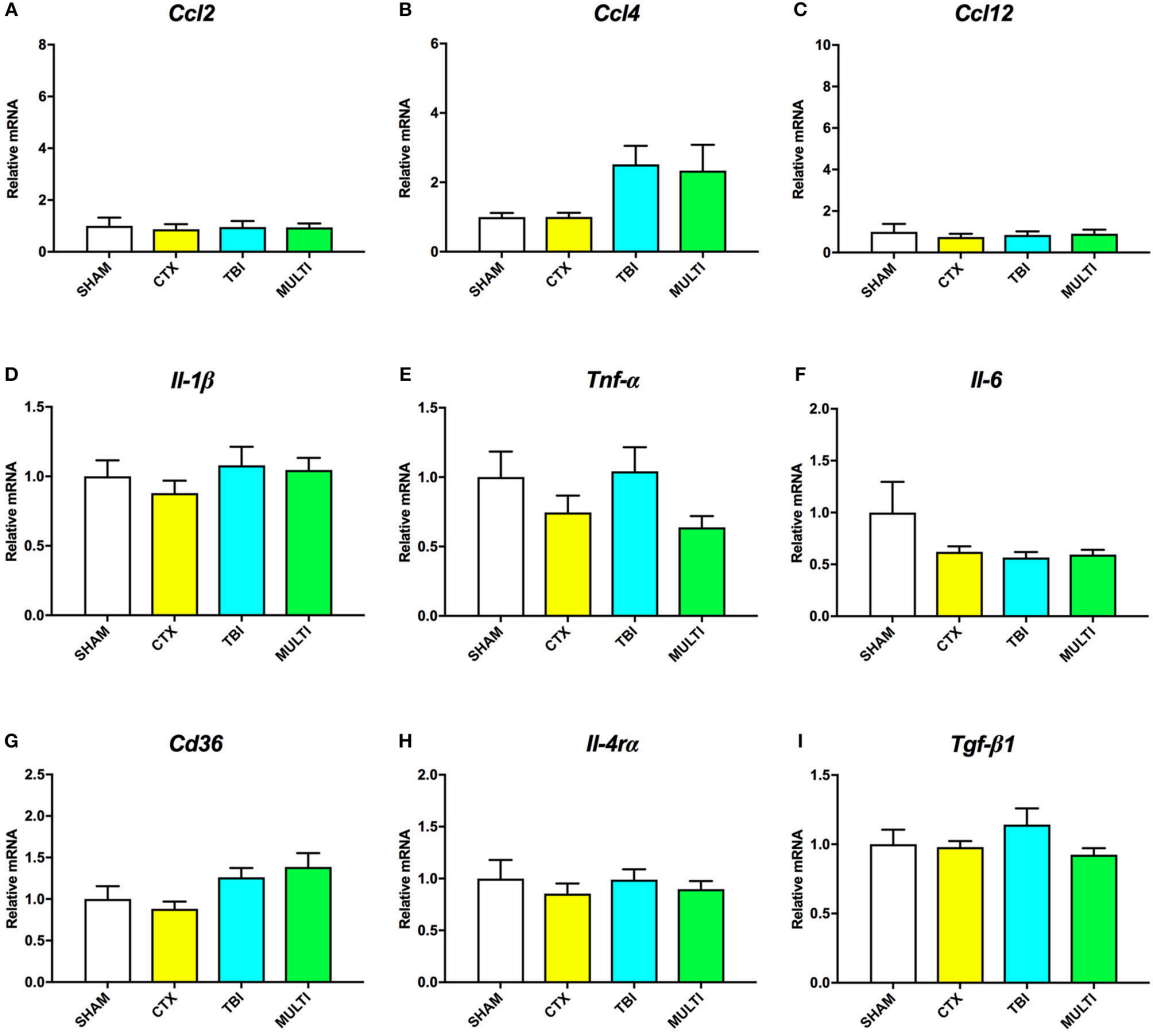
mRNA levels of pro- **(A–F)** and anti-inflammatory **(G–I)** cytokines in the ipsilateral cortex at 35 days post-injury. There was a significant effect of injury on *Ccl4* **(B)** and *Cd36* **(G)**. Although TBI and MULTI groups appeared to have similarly elevated levels of *Ccl4* and *Cd36, post-hoc* analyses did not reach statistical significance after Bonferroni corrections. There were no significant differences on mRNA levels of *Ccl2* **(A)**, *Ccl12* **(C)**, *Il-1*β **(D)**, *Tnf-*α **(E)**, *Il-6* **(F)**, *Il-4r*α **(H)**, or *Tgf-*β*1* **(I)**.

At 35 days post-injury, ANOVA indicated a significant effect of injury on the mRNA levels of *Ccl*4 [*F*_(3, 27)_ = 3.217, *p* < 0.05; Figure 5B] and *Cd*36 [*F*_(3, 27)_ = 3.124, *p* < 0.05; Figure 5G]. Although TBI and MULTI groups appeared to have similarly elevated levels of both *Ccl*4 and *Cd*36 relative to the SHAM and CTX groups, *post-hoc* analyses did not reach statistical significance after Bonferroni corrections. There were no significant differences on mRNA levels of *Ccl*2 (Figure 5A), *Ccl*12 (Figure 5C), *Il-1*β (Figure 5D), *Tnf-*α (Figure 5E), *Il-6* (Figure 5F), *Il-4r*α (Figure 5H), or *Tgf-*β*1* (Figure 5I). The TBI and MULTI groups were not significantly different on any of the 35 day RT-qPCR measures.

**Figure 6:**
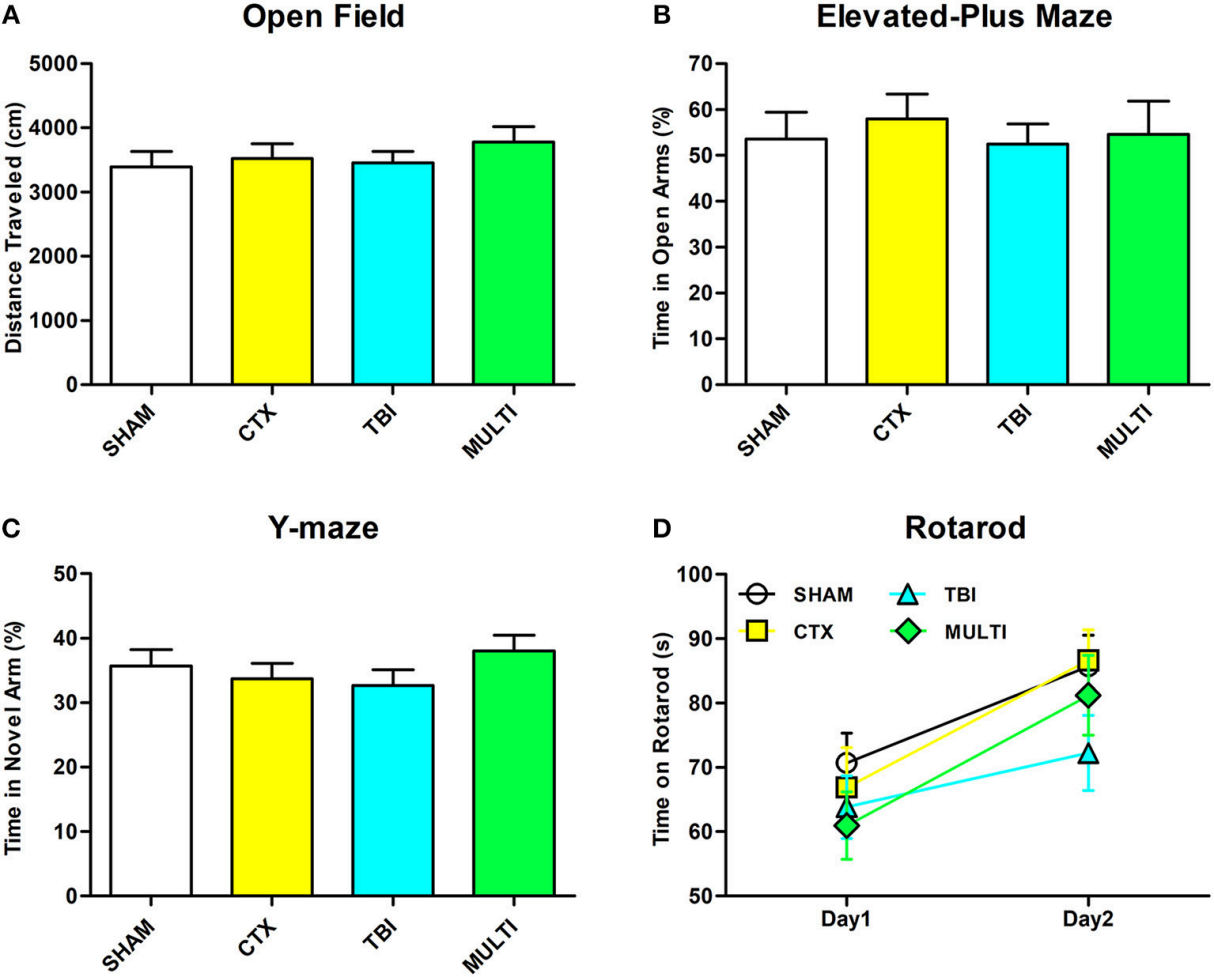
There were no significant differences between the groups on any of the behavioral measures. **(A)** Distance traveled in open field. **(B)** Time spent in open arm of the elevated plus maze. **(C)** Time spent in the novel arm of the Y-maze. **(D)** Time spent on rotarod.

There were no statistically significant findings between the experimental groups on any of the behavioral measures after a 30-day period of recovery after injury (Figure 6, *p* > 0.05).

## Discussion

This study investigated whether a concomitant muscle injury affected TBI pathophysiology and behavioral outcomes by combining mouse models of a closed-skull TBI and a CTX-induced muscle injury. Both groups that were given a TBI (i.e., isolated TBI and the MULTI) had worse acute injury measures (i.e., longer apnea, unconsciousness, and self-righting times); increased brain water content (i.e., edema); and elevated markers for astrogliosis (i.e., GFAP), neutrophils (i.e., MPO), and inflammatory chemokines. Overall, the isolated TBI and MULTI groups were not significantly different on any of the measures that were assessed, and there is little evidence from this initial study to suggest that a concomitant CTX muscle injury significantly impacts the aftermath of TBI.

These findings are in contrast to previous studies that have found that other forms of concomitant extracranial trauma can exacerbate TBI outcomes (3, 22, 41, 42). For example, a previous study from our laboratory found that, when compared to an isolated TBI, mice given a combined tibial fracture and closed-skull TBI had elevated edema at 24 h after injury; heightened neuroinflammation at 24 h and 35 days after injury; behavioral abnormalities at 35 days after injury; and enlarged ventricles and diffusion MRI abnormalities at 35 days after injury (3). Notably, these exacerbated outcomes in the tibial fracture + TBI mice were associated with a robust upregulation of the pro-inflammatory cytokine IL-1β in the injured cortex that was present at both 24 h and 35 days post-injury. Similarly, another previous study found that peripheral administration of IL-1β either 30 min or 24 h following TBI resulted in worse behavioral deficits and increased brain damage in rats (43). We therefore studied whether treatment with an IL-1 receptor antagonist (IL-1ra) beginning 1 h post-injury would improve outcomes in the combined tibial fracture + TBI model, and found that the IL-1ra treatment mitigated brain damage and neuroinflammation (5). As such, early IL-1β signaling appears to play a central role in the effects that an extracranial trauma can have on TBI. Consistent with this notion, in the current study the isolated TBI group and the CTX + TBI groups did not significantly differ on the measure of *Il-1*β mRNA levels–which may have contributed to the lack of other differences between these groups.

CTX is a protein kinase C-specific inhibitor that induces depolarization and contraction of muscle cells, and consequently damages the structure of cell membranes (28, 29). It is recognized as one of most common and reliable muscle injury models, and results in physiological events that occur in human muscle injury, including an inflammatory response (18, 21, 29). Indeed, the current study found that the gene expression of several inflammatory markers (i.e., CCL2, CCL4, G-CSFr, and TGF-β) indicative of muscle injury were elevated at 24 h post-injury (30). However, studies that have characterized the expression of various cytokines after CTX injury have found that IL-1β is not upregulated until 4–7days post-CTX (21, 30). On the other hand, IL-1β reaches peak levels within 24 h after tibial fracture in mice (44). As such, although CTX does result in a delayed increase in IL-1β, it may occur outside of the temporal window for IL-1β to alter TBI pathophysiology in a meaningful manner.

There are limitations with this study that should be considered when interpreting the findings and pondering future directions. In this study, outcomes were assessed at either 24 h or 30–35 days post-injury, which is consistent with our previous paper that investigated the effect of fracture on TBI (3). However, the pathophysiology of TBI, including neuroinflammation, has temporal complexities and future studies could employ additional recovery times to better characterize the potential effect of muscle injury on TBI. As discussed above, different forms of extracranial traumas (i.e., facture vs. muscle injury) have very different inflammatory profiles and temporal complexities that may affect TBI at different recovery times. This study also focused on neuroinflammation, however other physiological processes involved in TBI should also be explored. Furthermore, although no significant differences between the TBI and MULTI groups were found at 1 month post-injury, it is possible that differences may have eventually manifested if a longer recovery time was investigated. The current study was also limited to a single time-point for behavioral testing, and a limited number of tasks within that timeframe. Future studies could investigate functional outcomes at a number of acute, sub-acute and chronic recovery time-points and include additional behavioral tasks. The lack of behavioral deficits and minimal neuroinflammation in the mice given a TBI in the current study is likely due to the relatively mild nature of the model [i.e., in comparison to the more robust functional deficits and brain damage observed on other preclinical TBI models such as the fluid percussion injury and controlled cortical impact; (45)]. Indeed, in our prior study that examined the effects of fracture on TBI it was only the mice that were given the multitrauma that had significant behavioral deficits. Although the current TBI model did result in clear pathological changes, future studies could employ more severe models that consistently induce functional deficits, such as the fluid percussion injury or controlled cortical impact model (22–24). It may also be worthwhile for future studies to utilize other muscle injury models. Here we applied a CTX-induced injury as it is a well-characterized, reproducible, and commonly used model that involves a peripheral inflammatory response. However, future studies could examine whether a more traumatic model (e.g., crush injury), may have different effects on TBI outcomes. Indeed, a study comparing different muscle injury models found that although they all involved a form of an inflammatory response, some of the inflammatory factors and their trajectories differed between models (29).

In conclusion, TBI is a highly heterogeneous condition and often involves multitrauma. Clinical studies suggest that peripheral injuries are associated with an increased risk of mortality and functional deficits in TBI patients, particularly when severe extracranial injuries are combined with mild to moderate TBI (22). Previous studies in rodents have found that forms of concomitant extracranial inflammatory insults, such as a long bone fracture, can exacerbate TBI outcomes (3, 46). Contrary to these findings, here we found that mice given a TBI and concomitant CTX muscle injury did not differ from mice given an isolated TBI on any of the outcomes that were measured. These initial findings suggest that a muscle injury does not significantly impact TBI, and that the effect of extracranial trauma on TBI may be trauma specific. However, future studies that employ different TBI and muscle/extracranial injury models, as well as different time points and outcomes, should be conducted to allow for more concrete conclusions.

## Author Contributions

SS, SM, BS, TO, and JC conceptualized and designed the study. RB, CP, SM, and SS completed all injuries. MS and DA completed behavioral testing and post-mortem analysis. All authors contributed to the interpretation of the data and writing of the manuscript. All authors read and approved the final manuscript.

### Conflict of Interest Statement

The authors declare that the research was conducted in the absence of any commercial or financial relationships that could be construed as a potential conflict of interest.
